# Acute estradiol and progesterone therapy in hospitalized adults to reduce COVID-19 severity: a randomized control trial

**DOI:** 10.1038/s41598-024-73263-5

**Published:** 2024-09-30

**Authors:** Dragana Lovre, M. M. Fahd Qadir, Kristin Bateman, Leia Y Saltzman, Mya Sherman, Franck Mauvais-Jarvis

**Affiliations:** 1 Section of Endocrinology and Metabolism, John W. Deming Department of Medicine, Tulane University School of Medicine, New Orleans, LA 70112 USA; 2 Section of Endocrinology, Department of Medicine, Southeast Louisiana Veterans Health Care System, New Orleans, LA 70119 USA; 3 Tulane Center of Excellence in Sex-Based Biology & Medicine, New Orleans, LA 70112 USA; 4 Section of General Internal Medicine and Geriatrics, John W. Deming Department of Medicine, Tulane University School of Medicine, New Orleans, LA 70112 USA; 5 Tulane University School of Social Work, New Orleans, LA 70112 USA; 6 Institutional Review Board - Health Science Research, University of Virginia, Charlottesville, VA 22908 USA

**Keywords:** Estrogen, Estradiol, Progesterone, COVID-19, Immune tolerance, Inflammation, Sex differences, Immunology, Endocrinology

## Abstract

COVID-19 outcomes are less severe in women than men suggesting that female sex is protective. The steroids estradiol (E2) and progesterone (P4) promote anti-inflammatory immune responses and their therapeutic use for COVID-19 has been under investigation. The aim of the study was to evaluate the efficacy of a short systemic E2 and P4 combination in mitigating COVID-19 severity in hospitalized men and women. In a phase 2, single center, double blind, randomized placebo-controlled trial, ten male and female participants hospitalized for COVID-19 with scores 3–5 on the 9-point WHO ordinal scale were randomized to receive either (1) E2 cypionate (5 mg, IM) and micronized P4 (200 mg, PO), or (2) placebo-equivalent, in addition to standard of care (SOC). The primary outcome was the proportion of patients whose WHO scores improved to 1–2 on the day of discharge. Secondary outcomes included length of hospital stay (LOS), days on oxygen therapy (DOT), readmission rates (RR), adverse events (AEs), and change in circulating biomarkers using untargeted proteomics and cytokine profiling. There were no significant changes between the groups in primary outcome, LOS, DOT, RR or AEs. The E2P4 group exhibited a decrease in biomarker pathways of respiratory and gastrointestinal disease inflammation, infection by coronavirus, and immune cell trafficking and inflammatory response. A short-term E2P4 treatment in patients hospitalized for COVID-19 decreases biomarkers of inflammation. Considering the availability, low cost, and safety of E2 and P4, our results warrant additional studies to explore their effects in mitigating other viral pandemics. *Clinical Trial Registration* NCT04865029, ClinicalTrials.gov; (First trial registration 29/04/2021).

**Supplementary Information:**

The online version contains supplementary material available at 10.1038/s41598-024-73263-5.

## Introduction

During the coronavirus disease 2019 (COVID-19) pandemic, men exhibited more severe outcomes than women, suggesting that female sex is protective from severe COVID-19.^1–6^ Women exhibit heightened immune responses to viral infections compared to men,^7^ and the protective antibody responses to vaccinations are greater in women than men.^8^ The ovarian steroids 17β-estradiol (E2) and progesterone (P4) exhibit immunomodulatory and anti-inflammatory properties via estrogen and progesterone receptors expressed in immune cells.^7,9^ E2 and P4 decrease innate immune cell production of proinflammatory cytokines, enhance T cell anti-inflammatory responses and immune tolerance, and enhance B-cell production of antibodies.^7,10,11^ One study reported that young women using oral contraceptives had lower rates of COVID-19 hospitalization.^12^ Large observational cohorts have suggested that estrogen supplementation in postmenopausal women with COVID-19 decreases risk of dying from COVID-19.^13–15^ One pilot randomized controlled trial (RCT) reported that subcutaneous injection of P4 in addition to standard of care (SOC) could mitigate moderate to severe COVID-19.^16^ Here, we tested the efficacy of a short E2 and P4 combination therapy, in addition to SOC, in mitigating COVID-19 severity in hospitalized men and women.

## Methods

### Study implementation, recruitment, population, and eligibility criteria

Ten participants were recruited within 72 h of admission for COVID-19 by the medical staff of the Department of General Internal Medicine and Geriatrics at Tulane Medical Center (tertiary care academic hospital) (Table 1). Eligible subjects were enrolled by research coordinators to partake in a randomized scheme to receive either the 5-day active treatment or placebo equivalent in addition to SOC. The Research Pharmacist at Tulane Medical Center assigned participants to the intervention; the rest of the team and the subjects were blinded. Participants were enrolled between August and October 2021.

**Table 1:** Consort diagram and baseline characteristics by treatment group and by sex.

CONSORT Flow Diagram										
Bseline characteristics										
	E2P4 (*n* = 5)		Placebo-eq. (*n* = 5)			Men (*n* = 5)		Women (*n* = 5)		
Demographics	Mean	SD	Mean	SD	P value	Mean	SD	Mean	SD	P value
Age (mean, SD)	51.40	12.92	45.60	13.41	0.51	43.60	11.06	53.40	13.61	0.20
*Race (n) (C, AA)	1, 4		2, 3			2, 3		1, 4		
*Female Sex n (%)	3 (60)		2(40)			0 (0)		5 (100)		
Clinical										
BMI (mean, SD)	24.50	5.47	38.76	6.87	0.01	31.42	7.13	31.84	12.38	0.90
Diabetes (n)	1		1			2		0		
HTN (n)	3		2			3		2		
COPD (n)	1		0			0		1		
Obesity (n)	1		4			2		3		
CAD (n)	1		0			0		1		
HF, Liver Dz, Cancer (n)	0		0			0		0		
CCI (mean)	2.40		1.40		0.35	1.6		2.2		0.5
Biochemical										
AST (< 39units/L)	78.80	74.96	78.20	61.22	0.99	111.20	67.67	45.80	45.99	0.17
ALT (30-65units/L)	62.60	60.01	70.20	47.52	0.83	98.60	52.41	34.20	23.57	0.05
CRP (< 0.3 mg/dL)	12.23	8.58	6.87	3.12	0.54	8.53	6.97	8.23	1.98	0.90
D-dimer (0-0.59 mg/L)	1.62	1.35	0.59	0.19	0.32	1.25	1.08	0.53	0.27	0.20
Ferritin (23-338ng/mL)	1518	1547	714.12	657.32	0.47	1545.60	1337.43	486.23	200.81	0.20
WBCs (4.5–11 K/mL)	6.62	0.72	6.38	1.91	0.80	6.36	1.97	6.64	0.55	0.77
Platelets (160–420 K/mL)	260.00	66.49	157.80	39.88	0.02	230.40	89.71	187.40	57.75	0.40
Neutrophils # (2.34-7.0)	4.84	0.72	4.37	1.51	0.55	4.72	1.63	4.50	0.49	0.79
Lymphocytes # (0.9–4.84)	1.25	0.18	1.43	0.70	0.60	1.07	0.40	1.61	0.45	0.08
NLR	3.96		3.85		0.93	4.81		2.80		0.13
Lung imaging										
With infiltrates (n)	4		4			5		3		
Normal (n)	1		1			0		2		

E2, estradiol; P4, progesterone; C, Caucasian; AA, African American; HTN, hypertension; COPD, Chronic obstructive pulmonary disease; CAD, Coronary artery disease; HF, Heart Failure; Dz, disease; CCI, Charleson Comorbidity Index; AST, aspartate transaminase; ALT, alanine transaminase; CRP, C-reactive protein; WBC, White Blood Cells; NLR, Neutrophil: Lymphocyte Ratio.

*Race and sex were self-reported. Sex is defined as sex assigned at birth.

All volunteers gave their informed written consent to participate in the study. Participants were men and women over 18 years of age who were hospitalized for COVID-19 (WHO ordinal scale score 3–5)^17^ (Table 2) confirmed by PCR at Tulane Medical Center in the Department of General Internal Medicine and Geriatrics. Participants had respiratory symptoms (fever, shortness of breath, or cough) or abnormal lung exam or chest imaging characteristic of mild to severe COVID-19 pneumonia and agreed to be placed on a prophylactic dose of anticoagulant to prevent of deep vein thrombosis (DVT) (if necessary). The participants were excluded if they were < 18 years of age, critically ill with COVID-19, taking hormones (testosterone, estrogen, or progesterone) or had estrogen-dependent malignancies, contraindications to estrogen or progesterone, severe liver or renal disease, history of blood clots including deep vein thrombosis related to clotting disease, or pulmonary emboli, or had planned major orthopedic surgery within six weeks. Detailed methods were described in a previous protocol publication in BMJ Open^18^.

**Table 2:** Study design and clinical outcomes.

Study design				
Primary outcome (Proportion of patients improving to scores 1 or 2 on the WHO scale on the day of discharge)				
		Subject ID	WHO score at baseline*	WHO score at discharge
Placebo-eq.				
	Men (*n* = 3)	1	4	2
		2	4	2
		3	4	8
	Women (*n* = 2)	4	4	2
		5	5	2
E2P4				
	Men (*n* = 2)	6	5	2
		7	4	2
	Women (*n* = 3)	8	3	1
		9	3	2
		10	4	2
Secondary outcomes				
	E2P4 (*n* = 5)	Placebo-eq. (*n* = 5)	Men (*n* = 5)	Women (*n* = 5)
Length of Stay (LOS) in days (mean (SD))	7.2 (4.09)	10.20 (7.53)	12.20 (6.69)	5.20 (1.92)
Days on Oxygen Therapy (DOT) (mean (SD))	4.40 (6.11)	9.80 (8.04)	11.40 (7.89)	2.80 (3.42)
Required Oxygen treatment (n (%))	4 (80)	5 (100)	5 (100)	4 (80)
Noninvasive oxygen device (n (%))	1 (20)	4 (80)	3 (60)	2 (40)
Invasive mechanical ventilation (n (%))	0 (0)	1 (20)	1 (20)	0 (0)
Discharged on Oxygen (n (%))	1 (20)	3 (60)	3 (60)	1 (20)
Discharged to home (n (%))	5 (100)	4 (80)	4 (80)	5 (100)
ICU admission (n (%))	0 (0)	1 (20)	1 (20)	0 (0)
**Readmission for COVID within 60 days (n)	0	0	0	0
Death (n)	0	1	0	1
**Vaccination prior to infection (n)	2	1	1	2

*Only Subjects with mild to moderate COVID-19 (WHO category 3, 4 or 5) were enrolled in the study.

World Health Organization (WHO) 9-point Ordinal Scale for Clinical Improvement score. 0-Uninfected, No clinical or virological evidence of infection; 1-Ambulatory, No limitation of activities; 2-Ambulatory, Limitation of activities; 3- Hospitalized mild disease, Hospitalized, no oxygen therapy; 4-Hospitalized mild disease, Oxygen by mask or nasal prongs; 5- Hospitalized mild disease, Non-invasive ventilation or high-flow oxygen; 6- Hospitalized Severe Disease, Intubation and mechanical ventilation; 7-Hospitalized Severe Disease, Ventilation + additional organ support; 8-Dead, death.

**Readmissions and vaccinations were self-reported.

The goal was to recruit up to 120 participants hospitalized at Tulane Medical Center; however, due to unforeseen circumstances (regulatory delays, low case numbers, vaccine approval, oral antiviral medicine approval, and replacement of Delta strain by a less severe Omicron strain) we ended the study in January 2022 with 10 subjects completing the study.

### Primary outcome

For all randomized patients with baseline inclusion criteria and WHO 9-point ordinal scale scores 3 to 5, the primary efficacy end point is the proportion of patients who improve to scores 1 or 2 on the WHO ordinal scale (Table 2) through day of discharge.

### Secondary outcomes

For all randomized patients, the following secondary outcomes were assessed:

1. Length of hospital stay.
2. Duration of O2 supplementation.
3. Cause of death.
4. Readmission rates.
5. Serious Adverse Event (SAEs).
6. Change in Biological markers of inflammation, hypercoagulability and tissue injury were obtained as a part of SOC.
7. Change in Biological markers via cytokine panel and untargeted proteomics studies.

There were two sets of collected biomarkers: (1) Biomarkers obtained by physicians treating the patients during the hospitalization as a part of standard of care (SOC) practices, and (2) biomarkers obtained by the study team at randomization and at the end of the five-day treatment period. The SOC biomarkers were obtained from all subjects at the randomization, but not all participants had a blood draw before discharge, so our SOC biomarkers are only shown at baseline in Table 1. The study-specific serum biomarkers from participants were assessed by Olink platform 48 cytokine panel and by untargeted proteomics at baseline and after treatments. Serum biomarkers for proteomics were analyzed at the Tulane Proteomics Core Facility. We had biomarkers from eight out of ten participants, as two female participants were discharged before the study team could obtain blood. The CONSORT reporting guidelines were used.

Participant demographics and clinical information can be found in Tables 1 and 2.

The CONSORT reporting guidelines were used^19^.

### Study procedures

Primary and secondary outcome measures were obtained from the subjects and the electronic medical records (EMR) Tables 1 and 2. Hospital laboratory measurements were taken from the SOC practice from the EMR and included cytokines, markers of inflammation, coagulation, and tissue injury upon admission. Clinical status was obtained from EMR and the subjects during the study in the hospital on daily basis, from EMR on Days 14 and 28, and from the subjects via telephone call on day 60. Additional study-specific blood samples were also obtained on the day of randomization and after treatment or upon discharge for the proteomics studies.

### Plasma cytokine profiling

Proteins were measured using the Olink^®^ Target 48 Cytokine panel* (Olink Proteomics AB, Uppsala, Sweden) according to the manufacturer’s instructions. The Proximity Extension Assay (PEA) technology used for the Olink protocol has been well described^20^ and allows 45 analytes to be analyzed simultaneously using 1 µL of each sample. In brief, pairs of oligonucleotide-labeled antibody probes bind to their targeted protein, and if the two probes are brought in proximity, the oligonucleotides will hybridize in a pair-wise manner. The addition of a DNA polymerase leads to a proximity-dependent DNA polymerization event, generating a unique PCR target sequence. The resulting DNA sequence is subsequently detected and quantified using a microfluidic real-time PCR instrument (Biomark HD, Fluidigm). Data is then quality controlled and normalized using an internal extension control and calibrators, to adjust for intra- and inter-run variation. The final assay readout is presented in pg/ml using a 4-Pl fit for absolute quantification. All assay validation data (detection limits, intra- and interassay precision data, etc.) are available on the manufacturer’s website (www.olink.com). Expression profiles of individual cytokines were plotted using GraphPad Prism v9 software, while global inter-sample expression relationships were plotted using the heatmap.2 function in R (see *Quantification and Statistical Analyses*).

### Plasma untargeted proteomics

The plasma samples were first processed by protein depletion resin (Thermo Scientific) to remove fourteen high-abundance proteins. Then the treated plasma sample was mixed with an equal volume of lysis buffer (40% trifluoroethanol, 50 mM Tris-HCl, 10 mM dithiothreitol), reduced at 91 °C for 10 min, sonicated for 5 min, and then alkylated with 25mM iodoacetamide (final concentration) in the dark for 20 min. After drying using a Vacufuge (Eppendorf, Hamburg, Germany), the pellet was reconstituted in 50mM Tris-HCl and digested with trypsin at a 1:50 enzyme-to-protein ratio overnight at 37 °C. Peptides were acidified with trifluoroacetic acid (TFA), and 20 µg was loaded on SDB-RPS StageTip. The StageTip was wetted with acetonitrile and equilibrated with 30% methanol/1% TFA, and 0.2%TFA, respectively. Samples were washed and loaded into StageTip with 99% propanol/1% TFA and 0.2%TFA, respectively and eluted with 80% ACN/1% ammonium hydroxide. Eluted peptides were dried and reconstituted in 2% acetonitrile/ 0.1% formic acid for LC-MS/MS analysis. For spectral library generation, SCX tips were prepared similarly. Peptides were loaded and eluted in six buffers with varying concentrations of ammonium acetate and acetonitrile, along with 0.5% formic acid. Fractions were dried and reconstituted in 2% acetonitrile/ 0.1% formic acid for LC-MS/MS analysis. LC-MS/MS analysis was performed using a nano-LC system coupled to a Q Exactive HFX Orbitrap (Thermo Fisher Scientific). Samples were separated on a C18 analytical column with a gradient of acetonitrile in 0.1% formic acid. Data were acquired using DDA and DIA. All DDA data were processed and combined into a spectral library by Spectronaut to identify specific tryptic peptides that were present in reference proteomes. The DIA data was analyzed by Spectronaut using the spectral library generated by the DDA analysis. Quantification of identified peptides was calculated as the average of chromatographic fragment ion peak areas across all reference spectrum libraries. Pathway activation profiles from QIAGEN (see *Quantification and Statistical Analyses*) were used to define activated or suppressed cellular pathways and plotted using ggplot2 in R (coding repository is present in GitHub see KRT).

### Quantification and statistical analyses

For plasma cytokine profiling, IL1B and IL17A did not pass the assay quality control. The Olink Target 48 Cytokine panel results are presented as means ± SD and analyzed by Student’s t-test or one-way ANOVA, followed by a post-hoc Bonferroni test or Fisher’s LSD test when appropriate, with Graphpad Prism v9 software. *p* ≤ 0.05 was considered as statistically significant.

The untargeted proteomics data of all study participants were analyzed via normalized fold changes (FC) of E2P4 and placebo-eq. The formula used to calculate this normalized FC was: E2P4 FC (end of treatment/baseline)/placebo-eq FC (end of treatment/baseline). Protein FC data was used to perform an overrepresentation analysis (ORA) using Ingenuity pathway analysis (IPA, Qiagen) of diseases and biological functions. The expected causal effects between protein expression and ontological pathways are derived from the QIAGEN Knowledge Base. QIAGEN calculates pathway FDR-adjusted p-values and enrichment z-scores.

For baseline characteristics and clinical outcomes, a one-tailed t-test or two chi-square tests were conducted (as appropriate) to examine the mean differences between treatment groups and sex (men vs. women). In all cases, *p* ≤ 0.05 was considered as statistically significant.

## Results and discussion

### Clinical characteristics

Between August 2021 and October 2021, we recruited 10 participants (*n* = 5 male, *n* = 5 female) hospitalized for mild to moderate COVID-19. The study was terminated once COVID-19 vaccines were available, and the main SARS-CoV2 variant from Delta to Omicron resulted in a milder disease and decreased hospitalizations. Table 1 summarizes baseline sociodemographic and clinical characteristics of the cohort.

At baseline, participants had elevated levels of alanine transaminase (ALT), aspartate transaminase (AST), C-reactive protein (CRP), D-Dimer, and ferritin while white blood cell (WBC) count, neutrophils, lymphocytes, and neutrophil: lymphocyte ratio (NLR) were normal (Table 1). As seen in other studies, men trended toward higher transaminases (AST and ALT) and markers of inflammation (procalcitonin [PCT], ferritin, Interleukin 6 [IL6], NLR and CRP), while women exhibited higher platelets and lymphocyte counts.^21–26^ The placebo-eq. arm had a higher BMI than the E2P4 arm.

### Primary outcome

Participants assigned both to E2P4 and placebo-eq conditions exhibited similar regression from their baseline 3–5 scores on the 9-point World Health Organization (WHO) ordinal scale^17^ towards mild disease (score 1–2) on the day of discharge (Table 2).

### Secondary Outcomes

All clinical secondary outcomes, including LOS; DOT; percent of patients requiring oxygen treatment, a noninvasive oxygen device, invasive mechanical ventilation (IMV), and an intensive care unit (ICU) admission; and percent discharged on oxygen improved in the E2P4 vs. placebo arm but did not reach statistical significance (Table 2). Secondary outcomes were milder in women compared to men (irrespective of treatment arm), but this difference was also non-significant. SAEs were low in numbers and severity and significantly higher in the placebo-Eq. (16) vs. E2P4 group (6) (Table S1).

**Fig. 1:**
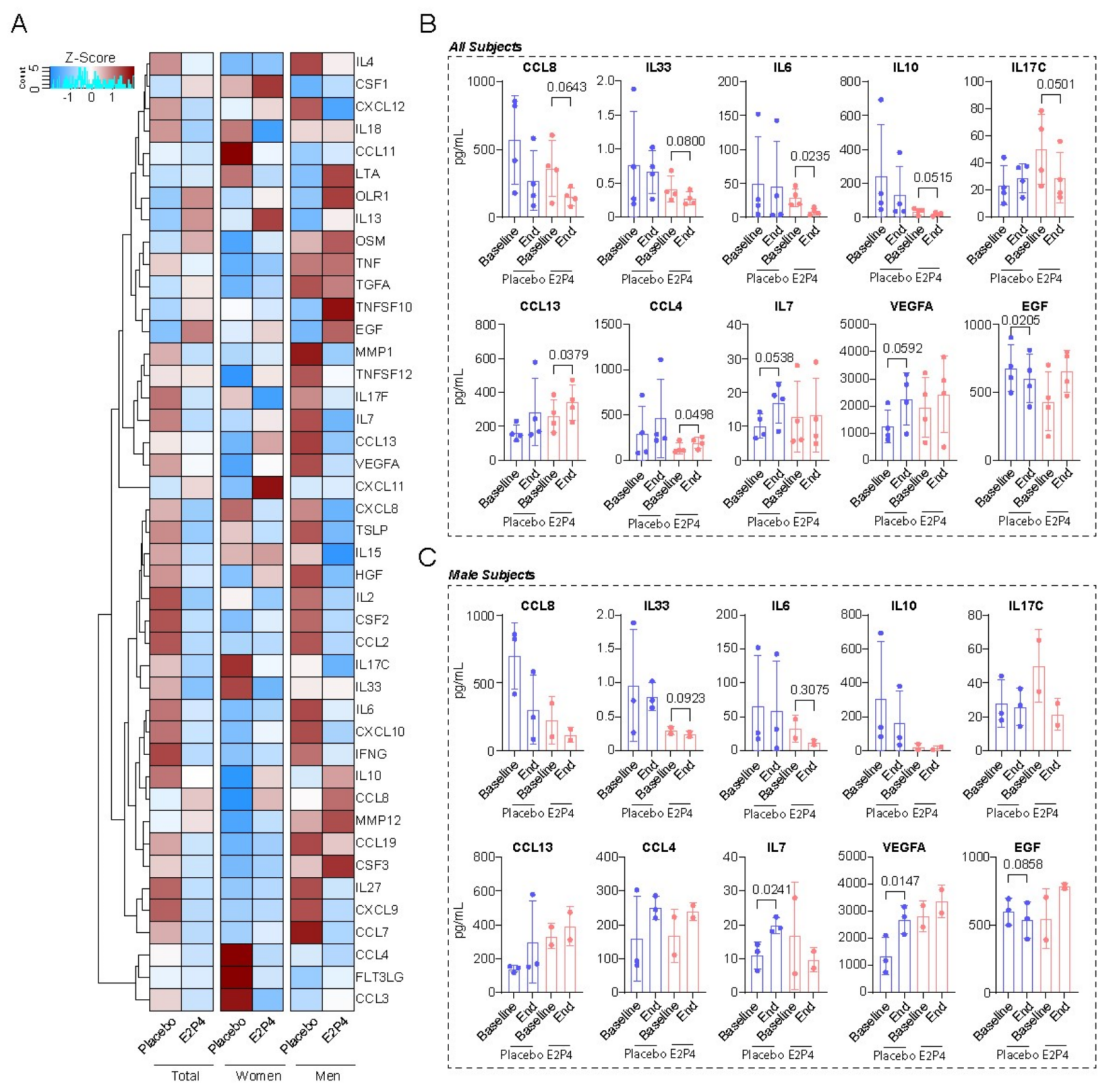
Effects of E2P4 on cytokines associated with inflammation. (**A**) Heat map of cytokine panel of 43 individual proteins by treatment group (before and after treatment) for (1) all subjects, (2) women alone, and (3) men alone. (**B**–**C**) Graphs showing expression profiles of select cytokines across all subjects (**B**) and male subjects (**C**). The color spectrum in (**A**) represents scaled Z-scores. Values in **B**–**C** represent means ± SD. Significant p-values (< 0.1) denoted on each graph. [C-C motif chemokine (CCL), interleukin (IL), pro-epidermal growth factor (EGF) and vascular endothelial growth factor A (VEGFA)].

Due to a limited number of serum samples from female subjects at the study’s end, we analyzed two groups: combined men and women, and men only. Compared to placebo-eq patients, E2P4 patients showed significantly decreased IL-6, showed a trend toward decrease in IL-33, IL-10, and IL-17 C, and had diminished IL-7 increase in both the combined and men-only groups (Fig. 1A–C). E2P4 patients also showed increased CCL13, CCL4, and VEGFA and were protected against decrease in EGF compared to placebo-eq in both groups (Fig. 1A–C).

IL-6, IL-7, and IL-17 C are involved in the acute inflammatory responses and acute lung injuries associated with infections like COVID-19.^27^ Circulating IL-6 concentrations correlate with COVID-19 severity^27–30^ and are associated with the development of long COVID-19.^31^ IL-7 and IL-10 are also associated with COVID-19 severity,^32^ and IL-17 C contributes to respiratory epithelial cell and mucosal immunity.^33,34^ Although IL-10 is traditionally considered anti-inflammatory, recent evidence suggests it has a detrimental role in COVID-19 severity.^35^ IL-33 is linked to pulmonary fibrosis after severe COVID-19,^36^ and IL-33 blockade with the anti-IL33 monoclonal antibody itepekimab improves lung function in asthma patients.^37^ Elevated VEGFA predicts adverse COVID-19 outcomes,^38^ and VEGFA may contribute to long COVID,^39^ so the effect of E2P4 in decreasing proinflammatory cytokines and VEGFA is expected to be anti-inflammatory and beneficial in preventing long COVID. It is unclear why E2P4 seemed to increase CCL13 and CCL4 in our study, as these chemokines are implicated in severe COVID-19 hyperinflammatory response.^40^ The effect of E2P4 on EGFR is also poorly understood, as EGFR is proposed to play a role in SARS CoV-induced pulmonary fibrosis.^41^ The effects of E2P4 on the rest of cytokines associated with inflammation is shown in Figure S1.

**Fig. 2:**
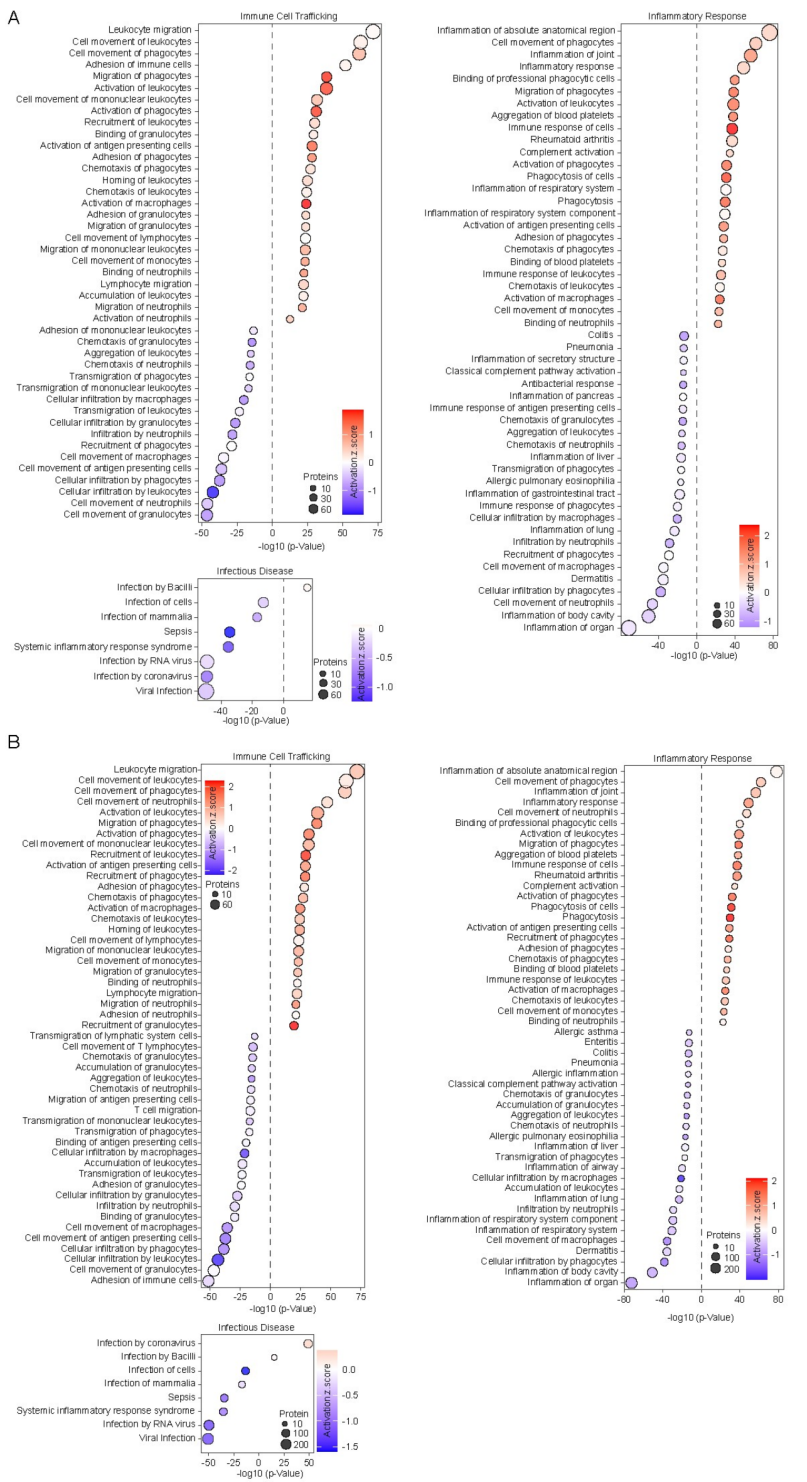
Effects of E2P4 on biological pathways involved in inflammation. (**A**–**B**) Bubble plot showing (1) immune cell trafficking, (2) inflammatory response and (3) infectious disease-associated pathway families for E2P4 vs. placebo-eq patients across: (**A**) All subjects. (**B**) All men. Pathway activation score scaled via Z-score (color intensity) and plotted with -log10 of the p-value (x-axis).

 We performed untargeted proteomics profiling of all study participants (Fig. 2 and S2, Table S2), which we analyzed using Ingenuity Pathway Analysis (IPA). E2P4 vs. placebo produced both a significant activation of immune cells and a decrease in infiltration of macrophages and neutrophils as well as markers of respiratory and gastrointestinal (GI) system inflammation (Fig. 2A and S2). E2P4 downregulates infectious disease (ID) pathways related to sepsis, systemic inflammation, infection by coronavirus and viral infection compared to placebo (Fig. 2A and B), meaning that the effect of E2P4 on these protein networks appears to be beneficial. We also observed an upregulation in pathways linked to various tumors including those affecting the liver, lung, and pancreas (Fig. 2A and S2). Interpreting these findings is challenging for multiple reasons: First, although the role of E2 and P4 in various cancers has been studied, the five-day treatment with E2P4 in the present trial is unlikely to increase tumor risk.^42–44^ Second, the prognostic and clinical relevance of these markers (Table S2) obtained from the Human Protein Atlas remain unclear and inconsistent across different proteins.

## Conclusions

A short systemic E2P4 treatment in hospitalized patients with mild to moderate COVID-19 improves systemic markers of inflammation and viral infection, though no significant clinical improvement was noted. Considering the availability, low cost, and safety of E2 and P4, our results warrant additional studies to explore their effects in mitigating other viral pandemics.

### Limitations of study

This study has several limitations. First, the small cohort size necessitates cautious interpretation of the results and their generalizability. Second, the use of folic acid as a placebo equivalent may have affected the regulation of the immune system potentially confounding the results. Lastly, due to the limited number of study participants, subject matching was challenging; in our investigation, the placebo-eq. group had a notably higher average BMI compared to E2P4 patients, and the E2P4 group had a higher average age than the placebo-equivalent group.

## Electronic supplementary material

Below is the link to the electronic supplementary material.

Supplementary Table 1-Adverse events and supportive therapies

Supplementary Table 2-Immune cell and cancer cell line expression across all participants and in men only.

Supplementary Figure S1

Supplementary Figure S2

Supplementary Legends

## Data Availability

The study data are available from the corresponding author upon reasonable request and with the permission of all contributing authors (Dragana Lovre, M.D., email dlovre@tulane.edu). The mass spectrometry proteomics data have been deposited to the ProteomeXchange Consortium via the Proteomics Identification database (PRIDE) partner repository with the dataset identifier PXD049133, and cytokine profiling was deposited with dataset identifier PXD049133. All data reported in this paper will be shared by the lead contact upon request.All original code has been deposited at Zenodo and is publicly available as of the date of publication: https://github.com/FMJLabTulane/E2P4_COVID_2024. DOIs. ^45,46^. Any additional information required to reanalyze the data reported in this paper is available from the lead contact upon request (Dragana Lovre, M.D., email dlovre@tulane.edu).

## Declarations

### Patient and public involvement

Patients and/or the public were not involved in the design, or conduct, or reporting, or dissemination plans of this research.
