# Supplementary material for: Acute estradiol and progesterone therapy in hospitalized adults to reduce COVID-19 severity: a randomized control trial
Source: Sci Rep. 2024 Sep 30;14:22732. doi: 10.1038/s41598-024-73263-5 (PMC11442588; doi:10.1038/s41598-024-73263-5)
Supplement: Supplementary file 2 — Supplementary Table 2-Immune cell and cancer cell line expression across all participants and in men only. [file 41598_2024_73263_MOESM2_ESM.docx]

| **Table S1. Adverse events and supportive therapies** | | | |  |  |
| --- | --- | --- | --- | --- | --- |
|  |  | **E2P4 (n=5)** | **Placebo-eq. (n=5)** | **Men (n=5)** | **Women (n=5)** |
| **Adverse events** |  |  |  |  |  |
| Septic shock (n (%)) |  | 0 (0) | 1 (20) | 1 (20) | 0 (0) |
| Hypoxia (n (%)) |  | 3 (60) | 5 (100) | 5 (100) | 3 (60) |
| Hypoperfusion (n (%)) |  | 0 (0) | 1 (20) | 1 (20) | 0 (0) |
| Cardiac arrest (n (%)) |  | 0 (0) | 1 (20) | 1 (20) | 0 (0) |
| Heart arrythmia (n (%)) |  | 1 (20) | 0 (0) | 1 (20) | 0 (0) |
| Cardiac enzyme elevation (n (%)) |  | 0 (0) | 1 (20) | 1 (20) | 0 (0) |
| Acute kidney injury (n (%)) |  | 0 (0) | 2 (40) | 1 (20) | 1 (20) |
| Acute liver injury (n (%)) |  | 2 (40) | 3 (60) | 4 (80) | 1 (20) |
| Pulmonary Embolism (n (%)) |  | 0 (0) | 0 (0) | 0 (0) | 0 (0) |
| Deep Venous Thrombosis (n (%)) |  | 0 (0) | 0 (0) | 0 (0) | 0 (0) |
| Thrombophlebitis (n (%)) |  | 0 (0) | 1 (20) | 1 (20) | 0 (0) |
| Total number of complications |  | 6 | 15 | 16 | 5 |
| **Other therapies** |  |  |  |  |  |
| Antibiotic treatments (n (%)) |  | 5 (100) | 5 (100) | 5 (100) | 5 (100) |
| Antiviral treatments (n (%)) |  | 2 (40) | 5 (100) | 4 (80) | 3 (60) |
| Systemic glucocorticoids (n (%)) |  | 5 (100) | 5 (100) | 5 (100) | 5 (100) |
| **Pulmonary therapies** |  |  |  |  |  |
| Oxygen treatment (NC) (n (%)) |  | 4 (80) | 5 (100) | 5 (100) | 4 (80) |
| Noninvasive oxygen device (n (%)) | | 1 (20) | 4 (80) | 4 (80) | 1 (20) |
| Noninvasive mechanical ventilation (n (%)) | | 0 (0) | 0 (0) | 0 (0) | 0 (0) |
| Invasive mechanical ventilation (n (%)) |  | 0 (0) | 1 (20) | 1 (20) | 0 (0) |
| NC, nasal cannula; Noninvasive oxygen device=NRB or HFNC, NRB, non-rebreather mask; HFNC, high-flow nasal cannula. | | | | | |
