# Supplementary figures and images for: Acute estradiol and progesterone therapy in hospitalized adults to reduce COVID-19 severity: a randomized control trial
Source: Sci Rep. 2024 Sep 30;14:22732. doi: 10.1038/s41598-024-73263-5 (PMC11442588; doi:10.1038/s41598-024-73263-5)

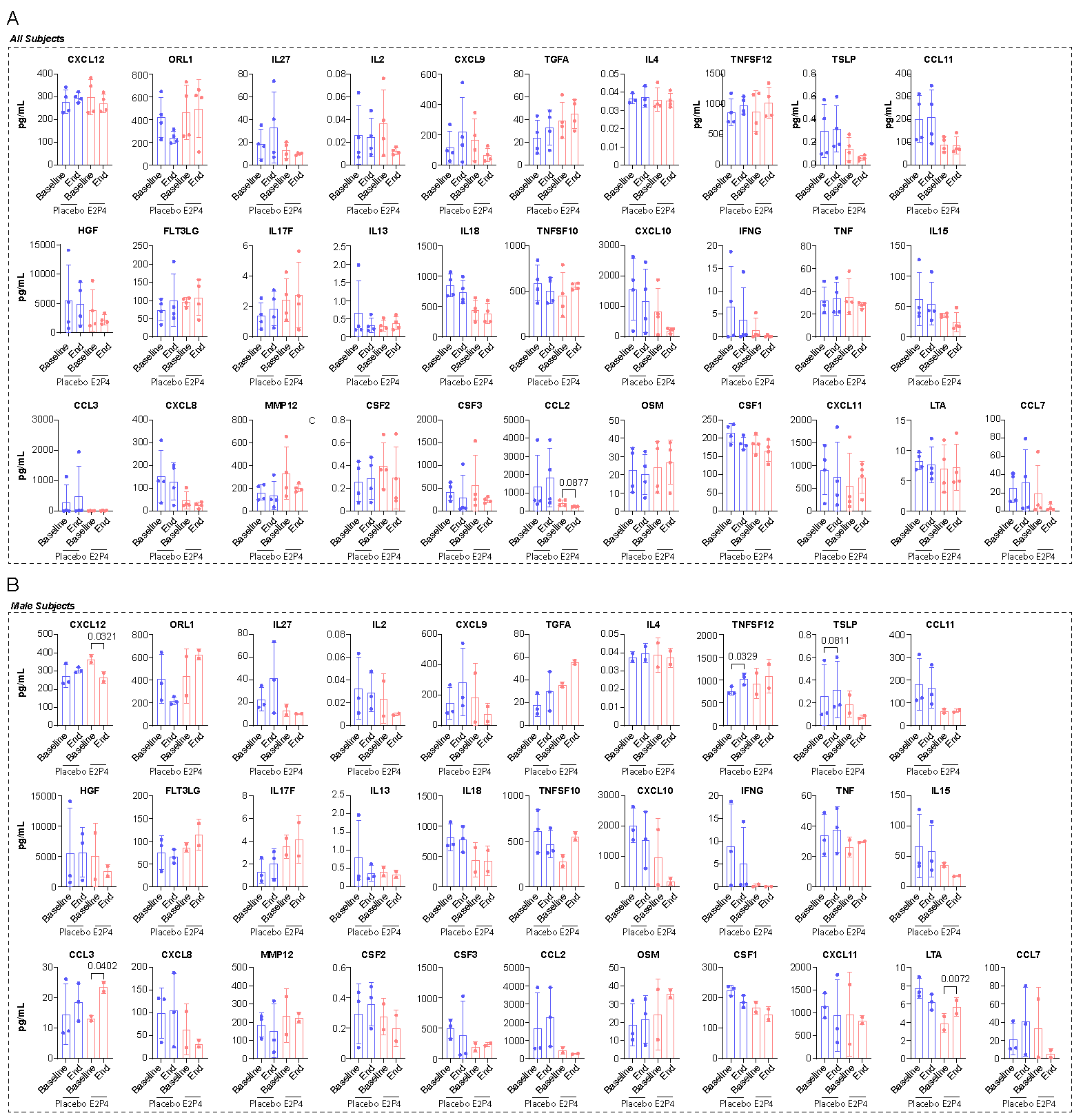

Supplement: Supplementary file 3 — Supplementary Figure S1 [file 41598_2024_73263_MOESM3_ESM.tif]

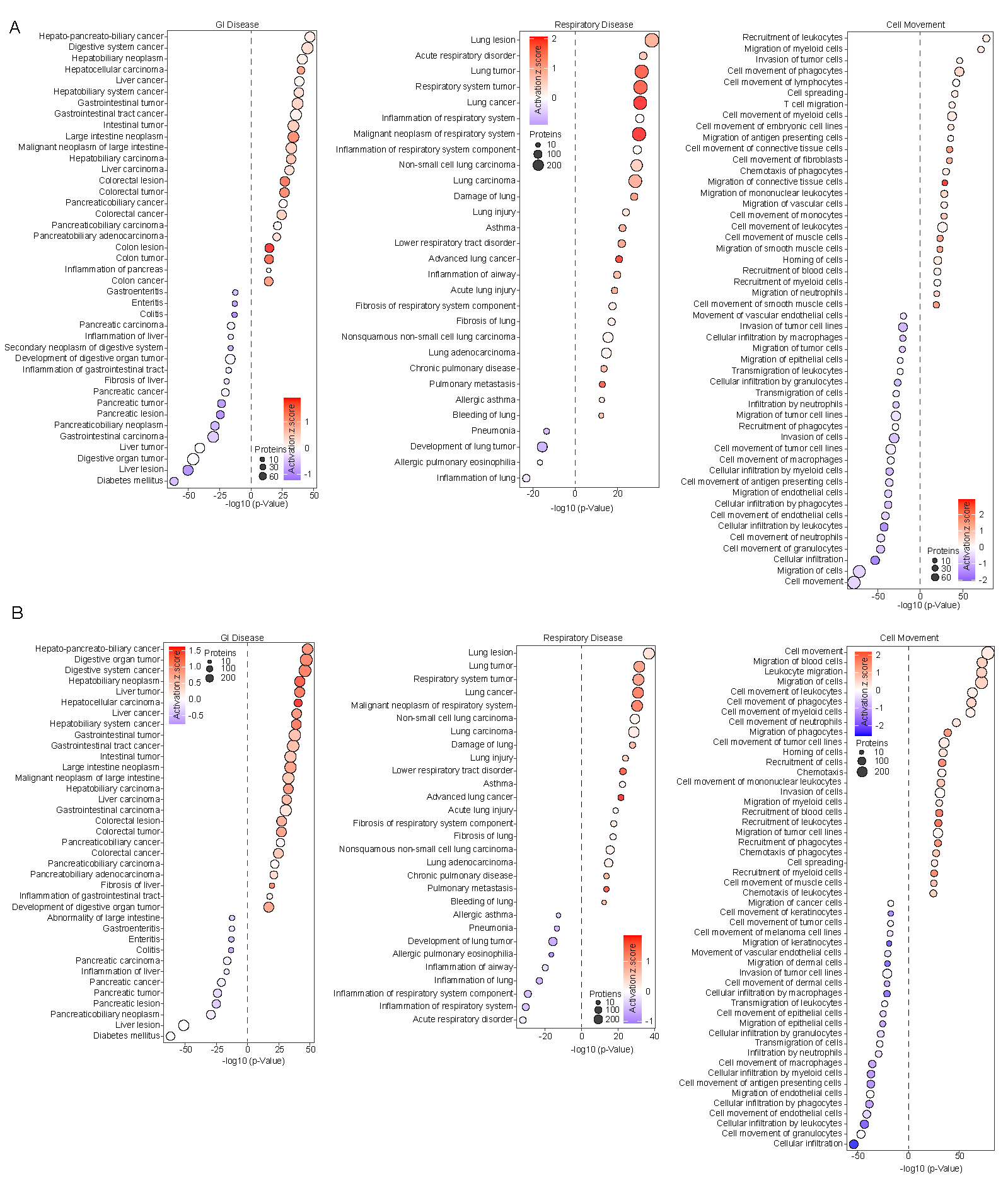

Supplement: Supplementary file 4 — Supplementary Figure S2 [file 41598_2024_73263_MOESM4_ESM.tif]
