# Supplementary material for: Acute estradiol and progesterone therapy in hospitalized adults to reduce COVID-19 severity: a randomized control trial
Source: Sci Rep. 2024 Sep 30;14:22732. doi: 10.1038/s41598-024-73263-5 (PMC11442588; doi:10.1038/s41598-024-73263-5)
Supplement: Supplementary file 5 — Supplementary Legends [file 41598_2024_73263_MOESM5_ESM.docx]

**Figure S1.** **Effects of E2P4 on cytokines associated with inflammation.**

(A-B) Graphs showing expression profiles of select cytokines across all subjects (A) and male subjects (B).

Values in A–B represent means±SD. Significant p-values (<0.1) denoted on each graph.

[Fms-related tyrosine kinase 3 ligand (FLT3LG), granulocyte colony-stimulating factor (CSF3), granulocyte-macrophage colony-stimulating factor (CSF2), interferon gamma (IFNG), interleukin (IL), lymphotoxin-alpha (LTA), macrophage colony-stimulating factor 1 (CSF1), oncostatin-M (OSM), tumor necrosis factor (TNF), tumor necrosis factor ligand superfamily member (TNFSF), thymic stromal lymphopoietin (TSLP), C-C motif chemokine (CCL), C-X-C motif chemokine (CXCL), eotaxin (CCL11), stromal cell-derived factor 1 (CXCL12), hepatocyte growth factor (HGF), interstitial collagenase (MMP1), macrophage metalloelastase (MMP12), oxidized low-density lipoprotein receptor 1 (OLR1), and protransforming growth factor alpha (TGFA)]

**Figure S2. Effects of E2P4 on biological pathways involved in inflammation.**

(A–B) Bubble plot showing 1) gastrointestinal (GI) disease, 2) respiratory disease and 3) cell movement associated pathway families for E2P4 vs. Placebo-eq across:

(A) All subjects.

(B) All men.M

Pathway activation score scaled via Z-score (color intensity) and plotted with -log10 of the p-value (x-axis).
